# Isocitrate Lyase and Succinate Semialdehyde Dehydrogenase Mediate the Synthesis of α-Ketoglutarate in *Pseudomonas fluorescens*

**DOI:** 10.3389/fmicb.2019.01929

**Published:** 2019-08-23

**Authors:** Azhar A. Alhasawi, Sean C. Thomas, Sujeethar Tharmalingam, Felix Legendre, Vasu D. Appanna

**Affiliations:** ^1^Department of Chemistry and Biochemistry, Laurentian University, Sudbury, ON, Canada; ^2^Department of Biology, Laurentian University, Sudbury, ON, Canada; ^3^Biomolecular Sciences Program, Laurentian University, Sudbury, ON, Canada; ^4^Northern Ontario School of Medicine, Laurentian University, Sudbury, ON, Canada

**Keywords:** isocitrate, γ-Amino butyric acid, succinate semialdehyde, α-Ketoglutarate, biofuel, enzymes, metabolic engineering

## Abstract

Glycerol is an important by-product of the biodiesel industry and its transformation into value-added products like keto acids is being actively pursued in order to improve the efficacy of this renewable energy sector. Here, we report that the enhanced production of α-ketoglutarate (KG) effected by *Pseudomonas fluorescens* in a mineral medium supplemented with manganese (Mn) is propelled by the increased activities of succinate semialdehyde dehydrogenase (SSADH), γ-aminobutyric acid aminotransaminase (GABAT), and isocitrate lyase (ICL). The latter generates glyoxylate and succinate two key metabolites involved in this process. Fumarate reductase (FRD) also aids in augmenting the pool of succinate, a precursor of succinate semialdehyde (SSA). The latter is then carboxylated to KG with the assistance of α-ketoglutarate decarboxylase (KDC). These enzymes work in tandem to ensure copious secretion of the keto acid. When incubated with glycerol in the presence of bicarbonate (${HCO}_{3}^{-}$), cell-free extracts readily produce KG with a metabolite fingerprint attributed to glutamate, γ-aminobutyric acid (GABA), succinate and succinate semialdehyde. Further targeted metabolomic and functional proteomic studies with high-performance liquid chromatography (HPLC), nuclear magnetic resonance (NMR) and gel electrophoresis techniques provided molecular insights into this KG-generating machinery. Real-time quantitative polymerase chain reaction (RT-qPCR) analyses revealed the transcripts responsible for ICL and SSADH were elevated in the Mn-supplemented cultures. This hitherto unreported metabolic network where ICL and SSADH orchestrate the enhanced production of KG from glycerol, provides an elegant means of converting an industrial waste into a keto acid with wide-ranging application in the medical, cosmetic, and chemical sectors.

## Introduction

The ability of microbial systems to transform chemical wastes and renewable biomass into value-added products has been widely exploited industrially. Citrate and glutamate are two common metabolites that are synthesized with the aid of microbes (Poblete-Castro et al., 2014; Radkov and Moe, 2014). The manipulation of metabolic networks is pivotal if these industrial processes are to be efficient. Genetic modification and abiotic modulation are central to the strategy aimed at reconfiguring metabolic modules designed to efficaciously obtain a desired product. α-Ketoglutarate (KG) is a keto acid of immense commercial interest due to its wide-ranging applications. Apart from being a pivotal chemical ingredient that participates in a variety of biological processes including protein synthesis, immune response, and anti-oxidative defense, it is also utilized clinically in wound healing, to treat chronic kidney deficiency and to decrease uremia (Tapiero et al., 2002; Bayliak et al., 2015). This keto acid is found in food flavoring, in cosmetics, and is utilized as a nutritional supplement (Wu et al., 2016).

The chemical synthesis of KG requires the participation of toxic ingredients and leads to the formation of undesirable products. For instance, it can be obtained from succinic acid and oxalic acid diethyl esters using cyanohydrines or by hydrolysis of acyl cyanide (Stottmeister et al., 2005). These multistep synthetic methods have a lot of drawbacks due to the use of hazardous chemicals such as cyanides and the generation of toxic wastes. Biotechnology offers a cleaner and more selective route to synthesize KG. In fact, numerous organisms have been shown to produce a variety of value-added products like succinate and butanol from glycerol (Poblete-Castro et al., 2019). Recently, the ability of the nutritionally versatile *Pseudomonas fluorescens* to produce copious amount of KG from glycerol in the presence of the micronutrient, Mn was reported (Alhasawi and Appanna, 2017). This affords a facile means to reprogram metabolic pathways compared to the procedures involving genetic transformation. The key participants in conversion of glycerol to KG are isocitrate dehydrogenase [ICDH-NAD(P)] and pyruvate carboxylase (PC). The overexpression of these two genes in numerous organisms has been routinely utilized to mediate the conversion of glycerol to KG (Yovkova et al., 2014; Beer et al., 2017). PC mediates the carboxylation of pyruvate into oxaloacetate, a precursor of citrate. ICDH then generates KG through the reductive carboxylation of isocitrate. In an effort to assess if any other metabolic networks are contributing to the enhanced production of KG in the Mn-supplemented cultures, a targeted metabolomic and functional proteomic study was undertaken to identify the metabolites and enzymes that orchestrate the formation of this keto acid. Manganese is known to be required for numerous enzymes to function effectively and can easily substitute for magnesium in biomolecules that depend on this divalent metal for optimal activity (Kehres and Maguire, 2003).

In this report, we demonstrate the ability of *P. fluorescens* to invoke an alternative network to produce KG from glycerol. Isocitrate lyase (ICL), succinate semialdehyde dehydrogenase (SSADH), fumarate reductase (FRD), GABA transaminase (GABAT), and α-ketoglutarate decarboxylase (KDC) appear to be the critical contributors to this KG-generating pathway. The pool of succinate that is supplemented by FRD and ICL contributes to the succinate semialdehyde budget. The synthesis of the latter is also aided by GABAT. KDC subsequently interacts with ${HCO}_{3}^{-}$ and SSA to produce KG. The significance of intact cells to secrete KG and the metabolic networks participating in this process are also discussed.

## Materials and Methods

### Microbial Growth Conditions and Cellular Fractionation

*Pseudomonas fluorescens* (ATCC 13525), obtained from the American Type Culture Collection, was grown and maintained in a mineral medium comprising Na_2_HPO_4_ (6 g), KH_2_PO_4_ (3 g), NH_4_Cl (0.8 g), MgSO_4_.7H_2_O (0.2 g) per liter of deionized water. Trace elements (1 ml) were added as well in concentrations as described previously (Anderson et al., 1992). The pH was adjusted using 2 N NaOH to 6.8. Glycerol (10% v/v: 1.37 M) from Sigma Aldrich (Oakville, Canada) was added to the medium as the sole source of carbon. This glycerol medium was dispensed in 200 ml aliquots in 500 ml Erlenmeyer flasks and inoculated with 1 ml of stationary phase (450 μg protein equivalent) of the control (without added Mn) *Pseudomonas fluorescens* culture. The media were supplemented with MnCl_2_ (50 μM) compared to the controls. This level of the divalent metal has been demonstrated to elicit the optimal production of KG (Alhasawi, 2018). The cultures were then aerated on a gyratory water bath shaker (model G76, New Brunswick Scientific) at 26°C and 140 rpm. The bacterial cells were isolated by centrifugation at stationary growth phases (40 h for control and 48 h for Mn-treated) and then resuspended in a cell storage buffer (CSB) consisting of 50 mM Tris-HCl, 5 mM MgCl_2_, and 1 mM phenylmethylsulfonylfluoride PMSF (pH 7.3). The cells were lysed by sonication using a Brunswick Sonicator on power level 4 for 15 s, 4 times, and within 5-min intervals. In order to obtain the cell-free extracts (ICE) soluble and membranous fractions, the cells were centrifuged for 3 h at 180,000 ×*g* at 4°C. The unbroken cells were initially removed by centrifugation at 10,000 ×*g* for 20 min. The Bradford assay was performed in triplicate to determine the protein concentration of both fractions using bovine serum albumin (BSA) as the standard (Bradford, 1976). Additionally, the cells were harvested at different time intervals by centrifugation at 10,000 ×*g* for 20 min. The bacterial pellets were treated with 1 ml of 1 N NaOH, and the biomass was measured with the aid of the Bradford assay. All comparative studies were performed with cells at the same growth phases, i.e., 40 h for the controls and 48 h for the Mn-treated cells. In order to confirm if the metabolic changes were indeed induced by the addition of Mn, control cells were added to Mn-treated media while cells isolated from the Mn cultures were incubated in control media and studied. To afford an accurate comparison, these cells were gathered at the same growth phase and following their incubation at 26°C for 6 h in a gyratory water bath, the cells were harvested as previously mentioned (Alhasawi et al., 2015b).

### Blue Native Polyacrylamide Gel Electrophoresis and Two-Dimensional SDS-PAGE

Blue native polyacrylamide gel electrophoresis (BN-PAGE) was utilized to assess the activities of various enzymes involved in the production of KG. To obtain optimal protein separation, 4–16% linear gradient gels were cast with the Bio-Rad MiniProteanTM2 (Hercules, CA) systems using 1-mm spacers. Sixty micrograms of soluble cell-free extract protein were loaded into the wells, and the gels were electrophoresed under native conditions (50 mM ε-aminocaproic acid, 15 mM Bis-Tris, pH 7.0, 4°C) at a final concentration of 4 mg of protein per milliliter. To aid in the solubilization, the membrane proteins were prepared with 1% (v/v) *n*-dodecyl β-D-maltoside. Blue cathode buffer [50 mM Tricine,15 mM Bis-Tris, 0.02% (w/v) Coomassie G-250 (pH 7)] was replaced with a colorless cathode buffer [50 mM Tricine, 15 mM Bis-Tris, (pH 7)] at 4°C when the running front was half-way through the resolving gel. The in-gel visualization of enzyme activity was carried out with the utilization of formazan precipitation. The gels were incubated in a reaction mixture containing the equilibration buffer [5 mM substrate, 0.5 mM cofactor with 0.2 mg/ ml of phenazine methosulfate (PMS) or DCPIP, and 0.4 mg/ml of iodonitrotetrazolium (INT)]. Succinate semialdehyde dehydrogenase (SSADH) activity was visualized using reaction buffer with 5 mM succinate, NAD(P)H and confirmed with the reverse reaction using 5 mM succinate semialdehyde, 0.5 NAD(P) (Auger et al., 2015; Kumar et al., 2015). Fumarate reductase (FRD) activity was also monitored in the gel using reaction buffer containing (5 mM fumarate, 0.5 mM NADH) (Thomas et al., 2016). Isocitrate lyase (ICL) activity was detected as described in (Auger et al., 2011) while the activity of α-ketoglutarate decarboxylase (KDC) was visualized by utilizing enzyme-coupled assays (Auger and Appanna, 2015). This was further confirmed with lead nitrate as well as the shift reagent (Yin et al., 2010). GABA aminotransminase (GABAT) activities were monitored by using (5 mM GABA, 5 mM KG, pyruvate, or oxaloacetate) (Choi et al., 1993; Han et al., 2012). In this instance, glutamate, pyruvate, oxaloacetate, or succinate semialdehyde were detected in the gel in either in the forward or reverse reactions. All reactions were stopped using destaining solution [40% methanol (v/v), 10% glacial acetic acid (v/v)] once the activity bands reached their desired intensity. To ensure equal loading, control cell-free extract (CFE) and stressed CFE were electrophoresed and stained with Coomassie blue. As a control, the activity of glutamate dehydrogenase (GDH), that did not show significant change in these cultures, was the internal control and was also probed as described (Alhasawi and Appanna, 2017). The activities ICL, FRD, SSADH, GABAT as well as KDC were further confirmed by incubating the activity bands with the appropriate substrates and monitoring the products by HPLC. Inhibitors like sodium arsenite (5 mM) and malonate (4 mM) were utilized to confirm the activity of SSADH and ICL, respectively (Singh et al., 2007; Pathare et al., 2013). Reactions were stopped using a destaining solution [40% methanol (v/v), 10% glacial acetic acid (v/v)] when the activity bands had attained the required intensity. Reactions performed without the addition of a substrate or cofactor or inhibitor in the reaction mixture ensured specificity. Densitometry was performed using Image J for Windows.

The activities of select enzymes were verified by spectrophotometric studies. Malate dehydrogenase (MDH) (2 mM malate and 0.5 NAD), α-ketoglutarate dehydrogenase (αKGDH) (2 mM KG, 0.5 NAD), and succinate semialdehyde dehydrogenase (SSADH) (2 mM succinate, 0.5 mM NADH) were monitored at 340 nm. DTNB [5,5-dithio-bis-(2-nitrobenzoic acid) was utilized to analyze succinyl-CoA synthetase (Williams et al., 1998)]. The reaction mixture contained 2 mM succinate, 1 mM ATP, 0.1 mM CoA in 25 mM Tris-HCl, and 5 mM MgCl_2_ buffer (pH 7.3). The decrease in absorbance due to consumption of CoA by DTNB ion was monitored at 10-s intervals for 10 min at 412 nm (*ε* = 13.6 mM^−1^·cm^−1^) (Williams et al., 1998; Bignucolo et al., 2013).

In-cell Western assays were modified from the Odyssey® Infrared Imaging System protocol document (Li-cor doc# 988-08599). Briefly, *P. fluorescens* were grown in control and Mn-treated cells. Equal amounts of cells (protein equivalent) were seeded in 96-well plates and fixed with 37% formaldehyde for 20 min at room temperature. Upon removing the fixing solution, the cells were washed thrice with phosphate buffered saline (PBS) (136.8 mM NaCl, 2.5 mM KCl, 1.83 mM Na_2_HPO_4_, and 0.43 mM KH_2_PO_4_ at pH 7.4). The PBS was then removed and the cells were soaked with PBS containing 0.1% tween-20 (v/v). Odyssey® blocking buffer was used as the blocking agent for 30 min. Primary antibody incubations occurred over a 1-h period with gentle shaking. Mouse monoclonal anti-SSADH (Gibson, Cornell University, NY) was diluted to a concentration of 1:200 in blocking buffer. Secondary antibodies consisted of donkey anti-mouse IR 680 (Li-cor; red) was diluted to 1:1,000. Incubation of the cells with secondary antibodies only allowed to account for any unspecific binding and to further confirm the loading of equal amount of cells (Auger et al., 2011).

### Real-Time Quantitative Polymerase Chain Reaction

Total RNA was extracted using TRI Reagent (Sigma), and RNA samples were then purified using the DNAse kit (Sigma) according to the manufacturer’s instructions. The DNAse treated RNA samples were reverse transcribed using random primers (Sigma), oligo dT (VWR), and M-MLV reverse transcriptase (Promega) in order to obtain complementary DNA (cDNA). Real-time quantitative polymerase chain reaction (RT-qPCR) was performed using QuantStudio5 (ThermoFisher). Each reaction was performed in 15 μl volumes containing 1 × Perfecta qPCR FastMix (Quanta Biosciences), 600 nM forward/reverse primers, and 10 ng cDNA. The cycling conditions were as follows: 1) 95°C for 2 min, 2) 95°C for 30 s (cDNA denaturation), 3) 57°C for 30 s (primer annealing), 4) 72°C for 30 s (template extension), 5) plate read and data collection, and 6) steps 2–5 repeated for 40 cycles. DNA melt curve analysis was performed at the end of each qPCR run to confirm specificity. Forward and reverse primer pair sequences for genes of interest were designed using Primer-BLAST offered by NCBI. The complete list of primer sequences can be found in Table 1. All primer pairs were subjected to stringent validation tests by plotting Ct values against cDNA serial dilutions. Primers with amplification efficiency between 90 and 110% [efficiency = 10^(−1/slope)^ − 1], and *R*^2^ value greater than 0.99 were considered validated and acceptable for qPCR analysis. All samples were normalized to two independent control housekeeping genes (rpoB and cpn60). The relative mRNA transcript level of each gene was reported according to the ΔΔ*C_T_* method as mRNA fold increase (Livak and Schmittgen, 2001).

**Table 1:** RT-qPCR primer sequences utilized in the present study.

Gene name	Genome ID (Gene location)	Sequence (5′ → 3′)	PCR product size (bp)	Annealing temp. (°C)
Isocitrate lyase (ICL)	NC_007492.2 (4,071,095–4,072,420)	Forward primer: CAACAACTCGTTCCGTCGTG	87	57
		Reverse primer: GGCGCGAAGTAGTCGATGTA		
Glutamine synthetase (GS)	NC_007492.2 (388,163–389,569)	Forward primer: AATACGGATCGAGGCGGAAC	92	57
		Reverse primer: CCACCAACTCCTACAAGCGT		
Succinate semialdehyde dehydrogenase (SSADH)	NC_007492.2 (225,316–226,758)	Forward primer: GTCGTCAGCTGATGTCGGAA	86	57
		Reverse primer: GTCGAACACGATGAATGGCG		
Pyruvate carboxylase (PC)	NC_007492.2 (6,316,963–6,318,771)	Forward primer: CCCACGGGTCTTCTTTCAGG	84	57
		Reverse primer: ACAAAGTCGGCTACTGGTCG		
DNA-directed RNA polymerase subunit beta (rpoB)	NC_007492.2 (5,714,594–5,718,667)	Forward primer: AGGCAAGGTCACTCCGAAAG	95	57
		Reverse primer: TGTCTTTAACGTCGCTGGCT		
Chaperonin 60 (Cpn60)	AY123661.1 (1–555)	Forward primer: AAAAACCTGTCCAAGCCATGC	81	57
		Reverse primer: GATGGAGCTGTCGGAGTTGG		

*Accession numbers for genes of interest were identified from NCBI database for *Pseudomonas fluorescens* Pf0-(NC_007492.2) or *Pseudomonas fluorescens* strain ATCC 13525 (AY123661.1). Forward and reverse primer sequences were designed using Primer-BLAST*.

### Metabolite Analysis

Metabolite levels were examined by high-performance liquid chromatography (HPLC) in which soluble cell-free extract (CFE) was taken promptly in order to reduce any degradation products and then boiled for 10 min to precipitate proteins prior to analysis. Quenching with 60% methanol afforded similar results (Alhasawi et al., 2015b). Samples of soluble CFE were injected into an Alliance HPLC equipped with a C18 reverse-phase column (Synergi Hydro-RP; 4 μm; 250 × 4.6 mm, Phenomenex) operating at a flow rate of 0.7 ml/min at ambient temperature. A Waters Dual Absorbance Detector was used as described in (Alhasawi et al., 2014). Electrophoretic activity bands were excised from the gel and placed in a reaction mixture containing 2 mM substrates, cofactors, and/or inhibitors. After 30 min of incubation, 100 μl of the sample was removed and diluted with Milli-Q water for HPLC analysis. The mobile phase containing 20 mM KH_2_PO_4_ (pH 2.9) was used at a flow rate of 0.7 ml/min at ambient temperature to separate the substrates and products, which were measured at 210 nm to detect carbonyl groups. To confirm the metabolite identity, biological samples were spiked with known standards and peaks were quantified using the Empower software (Waters Corporation). Also, to confirm that glycerol was being converted to KG, 4 mg of intact cells harvested from the Mn-supplemented cultures were incubated with 10% glycerol, 10 mM NH_4_Cl, and 10 mM ${HCO}_{3}^{-}$. The cells were stopped after 2 and 24 h by heating. Then, the metabolites were analyzed by HPLC. To identify the metabolic pathway involved in KG synthesis, the membrane CFE was reacted with glyoxylate, GABA, and ${HCO}_{3}^{-}$ in the presence of sodium azide. Similar experiments were performed with fumarate, ${HCO}_{3}^{-}$, and NADH as substrates.

### Nuclear Magnetic Resonance Studies

To establish if indeed enzymatic reactions were the source of succinate semi-aldehyde (SSA) and KG, the activity bands attributable to these enzymes were incubated sequentially with the ^13^C-labeled substrate(s) and the products were assessed. ^13^C-NMR analyses were achieved using a Varian Gemini 2000 spectrometer operating at 50.31 MHz for ^13^C. Samples were analyzed with a 5-mm dual probe (35 μ pulse, 1-s relaxation delay, 8 kilobytes of data, and 3,000 scans). Chemical shifts were established by comparing to standard compounds under equivalent conditions (Singh et al., 2009). The formation of SSA was first examined by incubating the excised band of SSADH from the membrane CFE of Mn cultures for 1 h in phosphate reaction buffer containing (2 mM succinate labeled ^13^C-1,4 and NADH). The reaction was stopped by removing the excised gel and heating at 60^°^C. Once the reaction was done, the products were examined by nuclear magnetic resonance (NMR). CFE was incubated with 2 mM GABA, 2 mM glyoxylate, and labeled $H^{13}{CO}_{3}^{-}$. The carboxylation of SSA into KG was monitored.

### Statistical Analysis

Data were expressed as means ± standard deviations (SDs). Statistical correlations of the data were tested for significance using the Student *t*-test (*p < 0.05, **p < 0.01) and all experiments were performed in triplicate in biological duplicate.

## Results

### Glycerol Metabolism and Ketoglutarate Production

**Figure 1:**
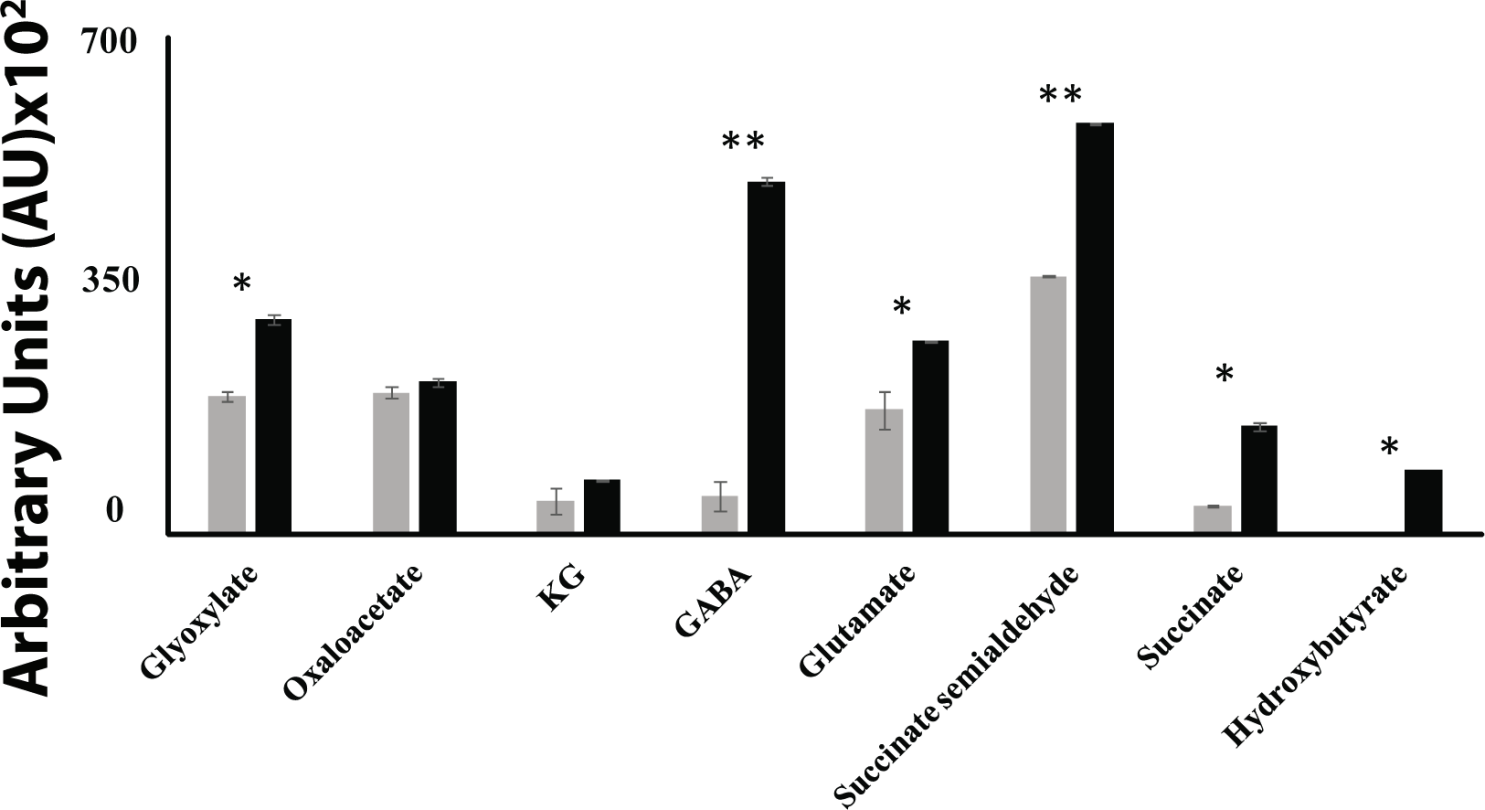
HPLC of metabolic profiles from control (gray bars) and Mn-treated cells (black bars) at stationary phase of growth. Data are presented as mean ± SD and are representative of three independent experiments (^*^p < 0.05; ^**^p < 0.01).

**Figure 2:**
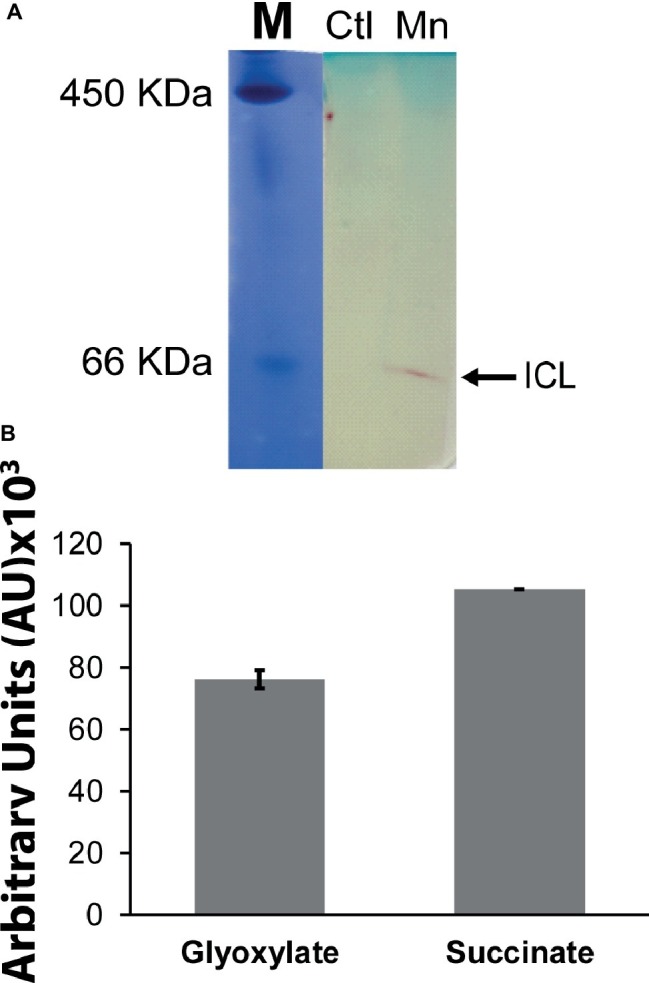
Succinate production mediated by isocitrate lyase (ICL). **(A)** In-gel enzymatic activity of ICL. Ferritin (450 KDa) and BSA (66 KDa) were used as molecular standards (M) with the Coomassie dye. **(B)** HPLC analysis of products following the incubation of excised activity band of Mn-treated cells with 2 mM isocitrate and 0.5 mM NAD for 30 min. ICL activity band was observed in the control after extended incubation periods. Gels are representative of three independent experiments. Ctl, control; Mn, Mn-treated.

**Figure 3:**
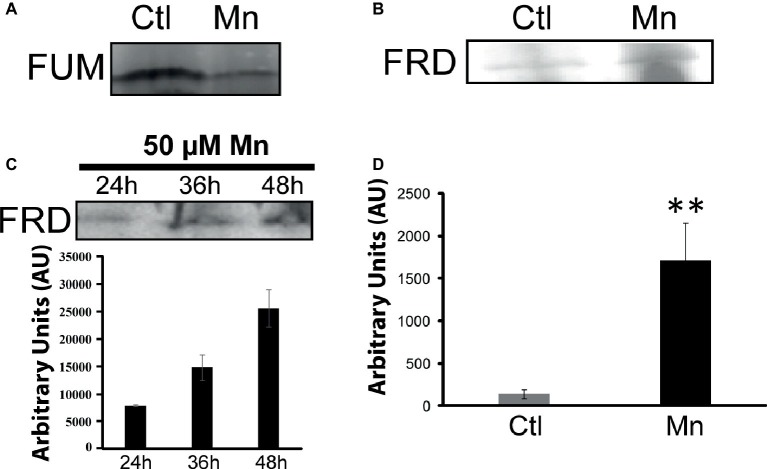
Fumarate metabolizing enzymes. **(A)** In-gel activity of fumarase (FUM). **(B)** Fumarase reductase (FRD) in-gel activity. **(C)** In-gel activity of FRD (Mn-cultures) at various time intervals with densitometric values. **(D)** Succinate analysis by HPLC of control and Mn excised bands **(B)** incubated in 2 mM fumarate and 0.5 mM NADH for 1 h. Gels are representative of three independent experiments (^**^p < 0.01). Ctl, control; Mn, Mn-treated.

When *P. fluorescens* is cultured in glycerol medium with Mn (50 μM), the microbe secretes copious amounts of KG compared to the control cells at stationary phase of growth (Alhasawi and Appanna, 2017). The soluble CFE isolated at the same growth phase had higher amounts of metabolites in the Mn-supplemented bacteria attributable to glyoxylate, succinate semialdehyde, GABA, 4-hydroxybutyrate, and succinate. While oxaloacetate levels were relatively similar, 4-hydroxybutyrate was detected only in the Mn-cultures (Figure 1). The succinate was increased approximately two-fold while GABA was nearly nine-fold higher compared to the control CFE. As succinate levels were markedly higher, this prompted us to evaluate how the homeostasis of this dicarboxylic acid was maintained and whether it was utilized in the biogenesis of KG, a metabolite found in elevated amount in the spent fluid. The presence of a glyoxylate peak in the CFE pointed to isocitrate lyase (ICL) as a possible mediator of this phenomenon. Indeed in the Mn-treated cells, the activity band indicative of this enzyme was intense. No ICL activity band in the control cells was discerned after an incubation period of 30 min (Figure 2A). The enzyme was inhibited by malonate (data not shown). When the ICL activity band was excised and treated with the isocitrate, peaks attributed to glyoxylate, and succinate were observed by HPLC (Figure 2B). In order to prove if indeed this increase in enzymatic activity was due to the presence of Mn in the medium, control cells were incubated in the Mn-media and Mn-grown cells were exposed to the control culture conditions. A reversal of ICL activity was detected (data not shown). Fumarate reductase (FRD) is another enzyme that can contribute to the succinate budget. While the activity of fumarase (FUM) was down in Mn-treated cells, the activity of FRD that mediates the conversion of fumarate into succinate with concomitant oxidation of NADH was elevated in the Mn-supplemented cultures (Figures 3A,B). The maximal enzymatic activity was observed at stationary phase of growth (Figure 3C). The activity band attributable to FRD generated a succinate peak when incubated with fumarate and NADH (Figure 3D). The synthesis of this dicarboxylic acid *via* KGDH did not appear to play a major role in the increased succinate observed in the cell-free extracts of the Mn-supplemented as this enzyme was markedly diminished. Also, succinyl CoA synthetase was markedly reduced in the Mn-treated cells (Table 2).

**Table 2:** Enzymatic activities in CFE from control and Mn-treated *P. fluorescens* at the same growth phase as monitored by spectrometry.

Enzymes	Control	Mn
Malate dehydrogenase (MDH)^a^	0.32 ± 0.07	0.22 ± 0.09
α-Ketoglutarate dehydrogenase (αKGDH)^a^	0.51 ± 0.14	0.44 ± 0.03
Succinate semialdehyde dehydrogenase^b^	0.45 ± 0.19	1.58 ± 0.33^*^
Succinyl-CoA synthetase^c^	0.12 ± 0.04	0.025 ± 0.01^*^

a *μmol NAD(P)H produced min^−1^ mg protein^−1^ as monitored at 340 nm (*n* = 3 ± standard deviation)*.

b *μmol NADH consumed min^−1^ mg protein^−1^ as monitored at 340 nm (*n* = 3 ± standard deviation)*.

c *μmol of CoA consumed min^−1^ mg protein^−1^ as monitored at 412 nm (*n* = 3 ± standard deviation)*.

* *Denotes a statistically significant difference compared with the control (*p* ≤ 0.05)*.

### Succinate Metabolism and Ketoglutarate Synthesis

**Figure 4:**
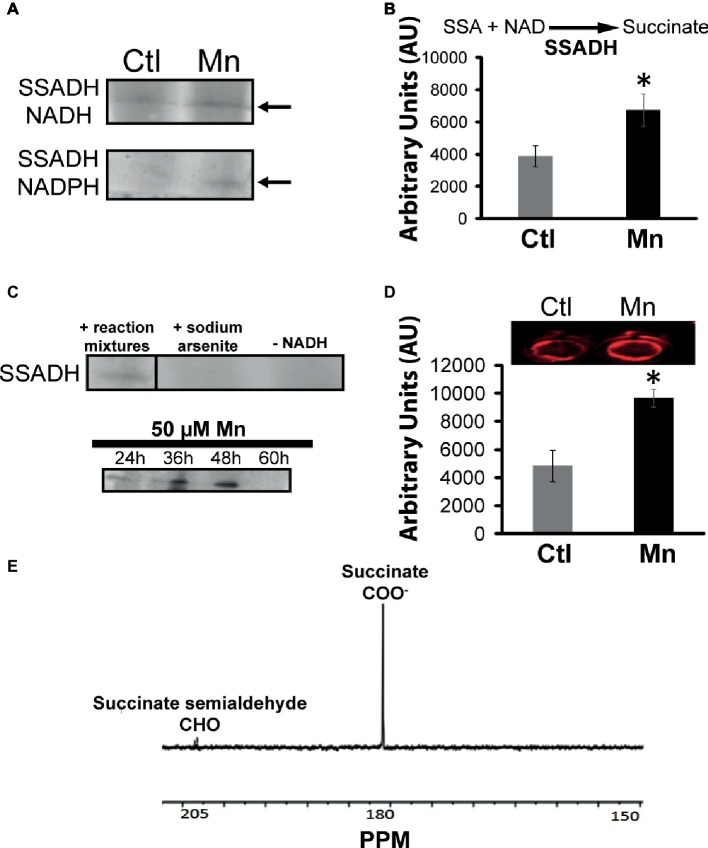
Analyses of succinate semialdehyde dehydrogenase (SSADH). **(A)** In-gel enzymatic activity of SSADH-dependent NADH and NADPH. **(B)** HPLC analysis of succinate production by SSADH activity band incubated in 2 mM succinate semialdehyde and 0.5 mM NAD after 1 h. **(C)** Activity of the SSADH, with 5 mM sodium arsenite (SSADH inhibitor) and without cofactor NADH (negative control) and at various time intervals in the Mn-cultures. **(D)** In-cell western blots with SSADH antibody (note: the control and Mn-treated CFE were exposed to secondary antibody only in order to account for any non-specific binding and to further confirm equal loading; no change in the intensity was observed (data not shown). **(E)** ^13^C NMR spectrum of SSADH excised band incubated in 1, 4 labeled succinate, and NADH after 1 h (note the aldehyde peak at 205 ppm). Gels are representative of three independent experiments; Ctl, control; Mn, Mn-treated. Densitometry was performed using Image J. ^*^p < 0.05.

Succinate can be consumed *via* succinate semialdehyde dehydrogenase (SSADH) and succinate dehydrogenase (SDH). The former converts succinate into succinate semialdehyde in the presence of NADH or NADPH. This enzyme was found to be elevated in the cells grown in the presence of Mn (Figure 4A). Excised activity bands readily generated succinate when incubated with succinate semialdehyde and NAD or NADP (Figure 4B). Sodium arsenite inhibited the activity of SSADH (Pathare et al., 2013). The influence of Mn on this enzyme was demonstrated by subjecting the Mn-treated cells to a control medium and the control cells to a Mn-supplemented medium. In this instance, a reversal of SSADH activity was observed (data not shown). It had a maximal activity at 48 h of growth, an incubation period coinciding with the optimal secretion of KG (Figure 4C). In-cell Western blot assays indicated an overexpression of this protein in cells harvested from the Mn-supplemented cultures compared to the controls (Figure 4D). The ability of the SSADH to produce SSA was confirmed by ^13^C NMR spectroscopy when the activity band incubated with labeled ^13^C-1, 4 succinate. The peaks indicative of aldehyde and carboxylic groups were detected (Figure 4E).

**Figure 5:**
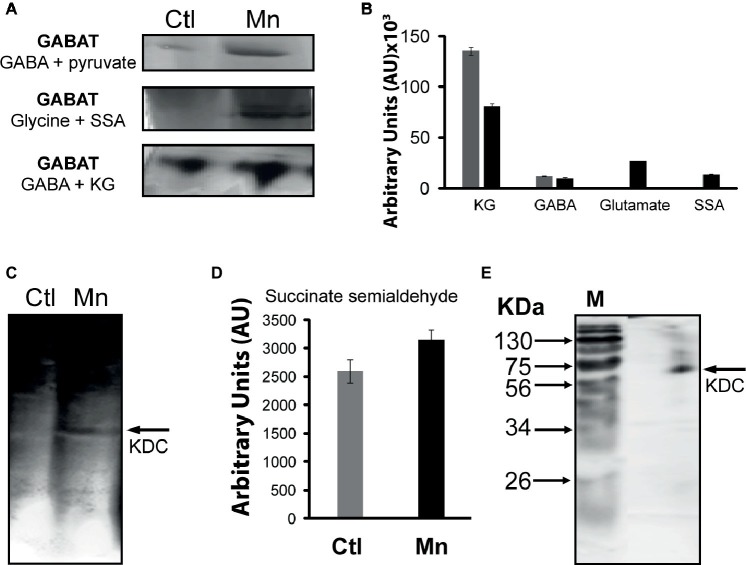
GABAT activity and succinate semialdehyde (SSA) synthesis. **(A)** In-gel activity of GABAT; metabolite analysis following the incubation of GABA and pyruvate with excised band. **(B)** In-gel activity of GABAT; product formation following the incubation of the excised band with GABA and KG. **(C)** α-Ketoglutarate decarboxylase (KDC) activity by lead nitrate precipitation. **(D)** HPLC analyses of succinate semialdehyde production from KDC excised band incubated in 5 mM of KG after 1 h. **(E)** 2D SDS gel of native KDC with Coomassie staining after 1D BN PAGE (M, molecular standards in KDa).

The presence of GABA in the CFE prompted the assessment of the contribution of GABA-metabolizing enzymes to SSA homeostasis. GABA aminotransaminase (GABAT) can mediate the conversion of keto acids and GABA into the corresponding amino acids and SSA. Activity bands corresponding to the transaminases converting GABA into SSA in the presence of glyoxylate, pyruvate, and KG were identified (Figure 5A). The formation of KG from glutamate and SSA that is effected by GABA-glutamate transaminase was also evident (Figure 5B). HPLC experiments with the excised bands helped established the substrate specificity of this enzyme (Figure 5B). Succinate semialdehyde can be converted into KG by the α-ketoglutarate decarboxylase (KDC), a biochemical transformation that necessitates the requirement of ${HCO}_{3}^{-}$ (Figure 5C). The activity band corresponding to this enzyme was detected in the membrane CFE by both formazan and lead carbonate precipitation. The latter detects the decarboxylation reaction while the former helps visualize the formation of SSA with INT and lactate dehydrogenase (LDH). Also, the enzyme was confirmed by incubating the KDC activity band with KG and SSA production was analyzed (Figure 5D). This enzyme was also verified by 1D BN-PAGE followed by two-dimensional (2D) SDS. The molecular mass of 56 KDa corresponded to that reported in the literature (Figure 5E; Zhang and Bryant, 2011).

### Enhanced Transcripts Responsible for Isocitrate Lyase and Succinate Semialdehyde Dehydrogenase Activity

**Figure 6:**
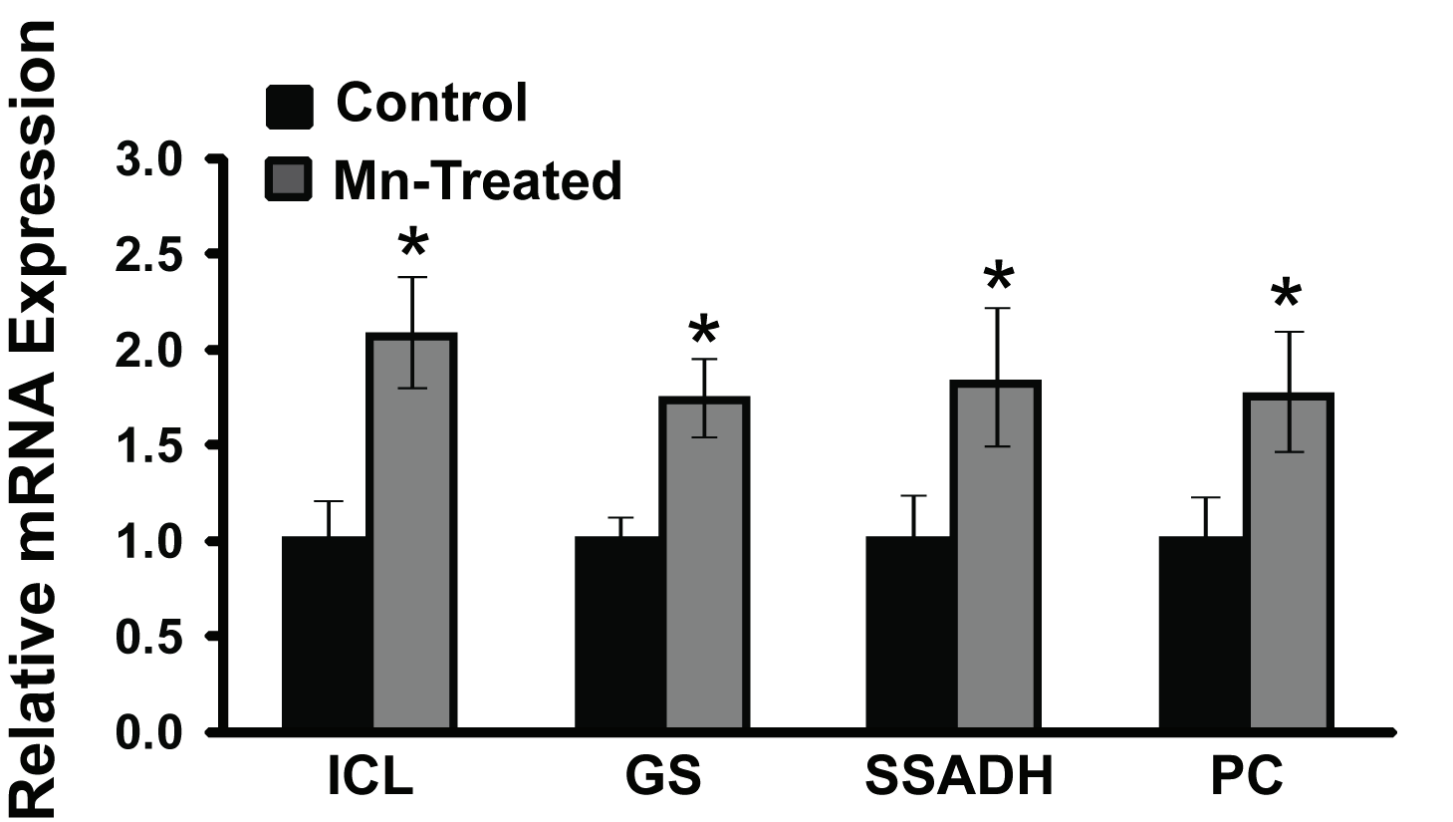
mRNA expression analysis of metabolic enzymes in Mn-supplemented cultures by RT-qPCR. mRNA transcripts of isocitrate lyase (ICL), glutamine synthetase (GS), succinate semialdehyde dehydrogenase (SSADH), and pyruvate carboxylase (PC) were determined relative to DNA-directed RNA polymerase subunit beta (rpoB) and chaperonin 60 (Cpn60) reference genes. Manganese-treatment (Mn-Treated) resulted in approximately 2-fold increased expression of ICL, GS, SSADH and PC. Results are representative of three independent experiments. Statistical significance was determined at *p* < 0.05 (^*^) using one-way ANOVA followed by Tukey’s *post hoc* analysis.

As the complete annotated genome of the organism utilized in this study is not currently available, the genetic information of *P. fluorescens* Pf0-1 (NC_007492.2) that is closely related to *P. fluorescens* 13525 was utilized. RT-qPCR primers were designed based on *P. fluorescens* Pf0-1 strain sequence (Table 1). Only mRNA expressions of some of the prominent enzymes are shown. RT-qPCR analysis revealed that Mn-treated cultures had increased mRNA expression of isocitrate lyase (2.07-fold), succinate semialdehyde dehydrogenase (1.82-fold), glutamine synthetase (1.74-fold), and pyruvate carboxylase (1.75-fold) compared to control cultures (*n* = 3; *p* < 0.05) (Figure 6). RT-qPCR primers designed for such enzymes like KDC and GABAT could not be validated and may have to await the complete genomic annotation of the strain used in this study.

### Identification of Metabolic Pathways: ^13^C NMR and High-Performance Liquid Chromatography Analyses of Cell-Free Extract and Intact Cells

**Figure 7:**
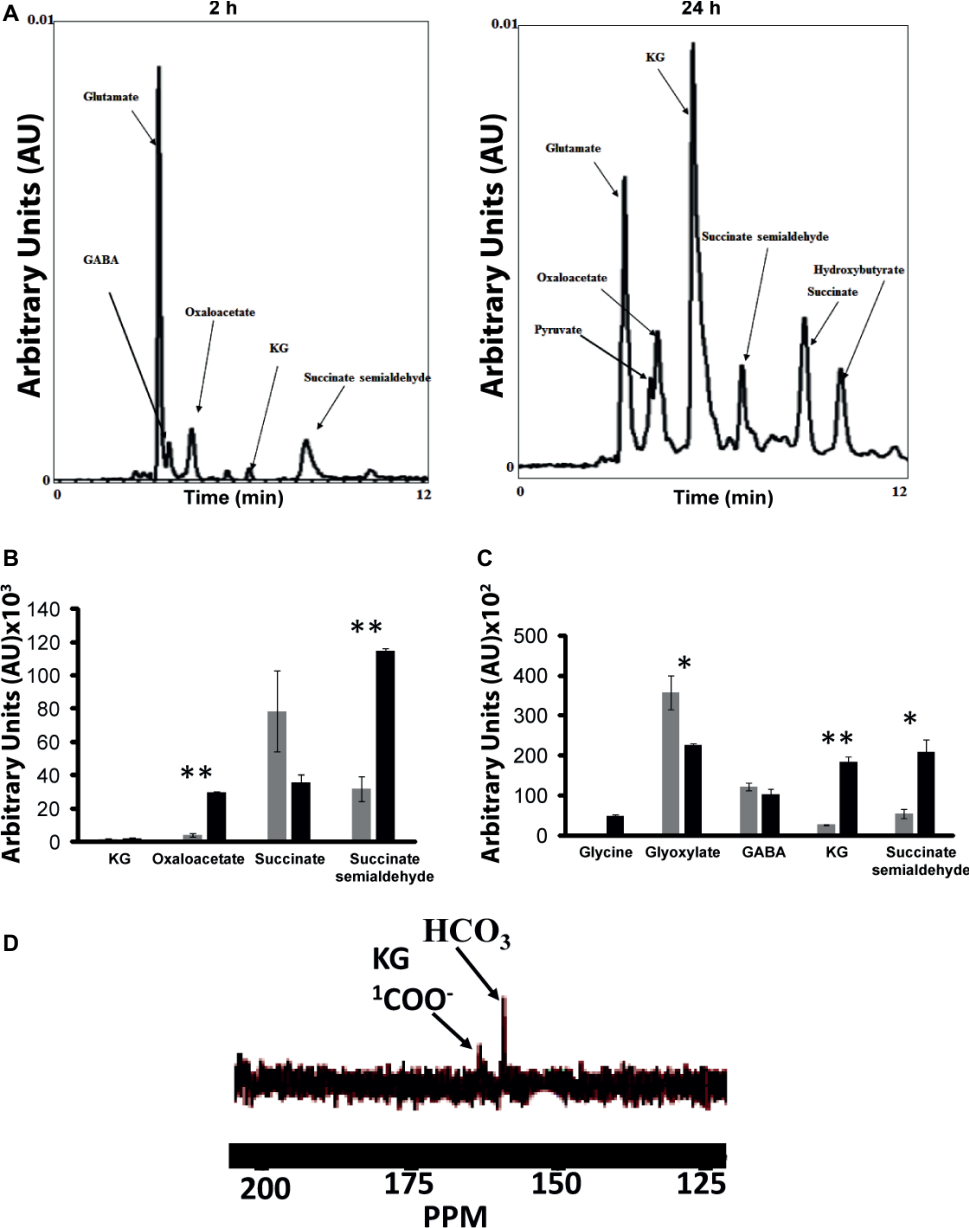
Experiments with intact cells and CFE. **(A)** HPLC analysis of metabolites when intact cells were incubated with glycerol and NH_4_Cl for 2 and 24 h, respectively. **(B)** Membrane CFE upon incubation with fumarate, NADH, and ${HCO}_{3}^{-}$. **(C)** Membrane CFE upon incubation with GABA and glyoxylate. **(D)** Membrane CFE upon incubation with GABA, glyoxylate, and labeled $H^{13}{CO}_{3}^{-}$ (note the COO^−^ of the product KG) ^*^p < 0.05; ^**^p < 0.01 (gray bar, control; black bar, Mn-supplemented).

As numerous enzymes that participated in the synthesis of various metabolites leading to the production of KG were identified by BN-PAGE, it became critical to evaluate if the intact cells and/or CFE would generate the metabolic fingerprint of this biochemical pathway. The incubation of the intact cells with glycerol, ${HCO}_{3}^{-}$, and NH_4_Cl yielded pyruvate, oxaloacetate, succinate, GABA, SSA, and KG peaks (Figure 7A). The membrane CFE revealed the presence of KG, oxaloacetate, SSA, and succinate in the presence of fumarate, NADH, and ${HCO}_{3}^{-}$ (Figure 7B). Also, when the membrane CFE was subjected to GABA, glycine, and ${HCO}_{3}^{-}$, SSA, glyoxylate, and KG were produced after 1 h of incubation (Figure 7C). In the presence of labeled $H^{13}{CO}_{3}^{-}$, glyoxylate, and GABA, a characteristic peak at 164 ppm corresponding to 1-COO^−^ group of KG was evident (Figure 7D). The keto acid was further confirmed by HPLC and dinitrophenylhydrazine (DNPH) assay (Middaugh et al., 2005). To limit the consumption of substrates *via* oxidation phosphorylation, these experiments were performed in the presence of sodium azide (5 mM) (Appanna et al., 2003).

## Discussion

The data in this study point to a pivotal role of succinate and SSA in the enhanced production of KG triggered by the micro-nutrient Mn when *P. fluorescens* is grown in a mineral medium with glycerol as the sole carbon source. The biosynthesis of KG from glycerol *via* the reductive decarboxylation of isocitrate, a reaction mediated by the enzyme ICDH (NAD/NADP) has been reported in numerous microbial systems (Yovkova et al., 2014). In this instance, PC provides oxaloacetate, the precursor that fuels this metabolic process. Insertion of multiple copies of genes harboring these two enzymes in the yeast, *Yarrowia lipolytica* resulted in 19% increase in the synthesis of KG. In *P. fluorescens,* the presence of Mn triggered a 5-fold augmentation in KG formation again propelled by PC and ICDH with the added participation of aspartate transaminase. This optimal synthesis of the keto acid is observed in the presence of 50 μM Mn. Pyruvate and oxaloacetate are the two important metabolites contributing to this process. Although Mn has been shown to modulate the synthesis of biopolymers, its participation in the enhanced formation of keto acids has hitherto not been reported (Amaya-Gomez et al., 2015; Alhasawi and Appanna, 2017). The activity of aconitase (ACN) that underwent a 2-fold increase would help replenish isocitrate, a key ingredient in this metabolic network (Holz et al., 2009). This tricarboxylic acid can undergo decarboxylation to directly generate KG with the aid of KGDH and can be cleaved by ICL into succinate and glyoxylate, moieties conducive to further manipulation into KG.

Although ICDH and PC are critical in the conversion of glycerol into KG as shown in our previous study (Alhasawi and Appanna, 2017), the current investigation reveals that succinate, GABA, and succinate semialdehyde are also pivotal intermediates orchestrating the production of the keto acid. The higher levels of the precursor metabolites compared the KG observed in the CFE may be due to fact that the latter is rapidly secreted in the spent fluid once synthesized. This may contribute to the increased production of KG and avert any feedback inhibition triggered by the intracellular accumulation of the keto acid. The enhanced activities of ICL and FRD contribute to the increased formation of succinate observed in the Mn-supplemented cultures. The former produces succinate following the cleavage of isocitrate while the latter adds to the budget of the dicarboxylic acid through its interaction with fumarate and NADH (Appanna et al., 2014; Thomas et al., 2016). The glyoxylate liberated when ICL reacts with isocitrate can be a source of malate that can be transformed into fumarate. FRD-fueled production of succinate is a common strategy invoked by numerous organisms dedicated to the secretion of this moiety (Appanna et al., 2014). It is important to note that ICL activity was barely evident in the control cultures and did not appear to be linked to malate synthetase (MS) as part of the glyoxylate shunt. When confronted by aluminum (Al) toxicity, *P. fluorescens* has been reported to overexpress ICL aimed at increasing the synthesis of oxalate, a metabolite known to sequester Al (Hamel et al., 2004; Auger et al., 2013; Alhasawi et al., 2015a). Hence, it is within the realm of possibility that this microbe utilizes ICL and FRD in tandem to enhance the formation of succinate, a metabolite that is then channeled toward the synthesis of KG.

The presence of peaks in the HPLC chromatograph characteristic of SSA and GABA would argue for the involvement of these moieties in the metabolic network mediating the enhanced production of KG. SSADH and GABAT are known to participate in the formation of SSA. These enzymatic activities were markedly elevated in the Mn-cultures. The transamination of glyoxylate and pyruvate in the presence of GABA liberates SSA and the corresponding amino acids. SSADH reduces succinate with the aid of either NADH or NADPH. These two enzymes participate in GABA metabolism in most organisms (Shelp et al., 1999; Dhakal et al., 2012). The amino-carboxylic acid that is a well-documented signaling molecule is also secreted during abiotic stress in plants and bacteria. It is involved in wound healing in plants, while in microbes, GABA contributes to acid tolerance (Su et al., 2011; Feehily and Karatzas, 2013; Bown and Shelp, 2016; Scholz et al., 2017). The SSA generated by the concerted action of SSADH and GABAT can readily fuel the synthesis of KG, a process mediated by either KDC or GABA-glutamate transaminase. These enzymes were indeed elevated in the cultures with added Mn. The carboxylation of SSA by KDC coupled with its transamination propelled by glutamate would provide an effective pathway to KG with the concomitant formation of GABA. The latter can become an ideal target of other GABAT in order to replenish the pool of SSA. KDC occurs in numerous microbes where various modified TCA cycle are operative (Sykes et al., 2015). KDC has a similar E1 component like KGDH but does not require any cofactors like co-enzyme A and NAD. It can decarboxylate KG to SSA and add ${HCO}_{3}^{-}$ to SSA to generate KG (Green et al., 2000). GABA-glutamate transaminase is a critical enzyme involved in modulating the homeostasis of GABA. Following its signaling role, GABA is eliminated with assistance of this enzyme (Dunn et al., 2009; Feehily and Karatzas, 2013; Mazzoli and Pessione, 2016). In this instance, it may contribute to enhanced KG formation. The data with the intact cells incubated with glycerol in the presence of ${HCO}_{3}^{-}$ and NH_4_^+^ would argue for such a possibility. The ability of the CFE to generate the metabolites participating in the biochemical pathway aimed at KG synthesis provides further evidence that confirms the in-gel activity assays of the corresponding individual enzymes. As Mn is known to participate directly in numerous enzymatic reactions and/or to substitute metals like Mg in a variety of proteins, it is not unlikely that the abundance of this micro-nutrient may be triggering this metabolic reprogramming dedicated to KG synthesis. ICL has been reported to be influenced by this divalent metal (Waldron et al., 2009). Furthermore, it may also be involved in promoting the expression of proteins that require Mn to function (Dunn et al., 2009). It has been well-established that lack of this mineral nutrient represses the transcription of the corresponding metallo-proteins (Hohle and O'Brian, 2014). They may help in the stabilization, modification, activation and elimination of transcription factors (Appanna and Preston, 1987; Rosch et al., 2009). These moieties can also modulate enzymatic activities (Karki et al., 2015; Gubert et al., 2016). Oxidative and nitrosative stress are known to trigger the production of keto acids in numerous organisms. Hence, metabolite synthesis triggered by metal nutrients and abiotic stress is not an uncommon occurrence (Mailloux et al., 2008; Lemire et al., 2010, 2017; Tharmalingam et al., 2017). Furthermore, Mn may be involved in the stabilization of mRNAs, an event that may be reflected in the increased transcripts attributable to the enzymes ICL, SSADH, and PC critical in the metabolic reprogramming observed in this study.

**Figure 8:**
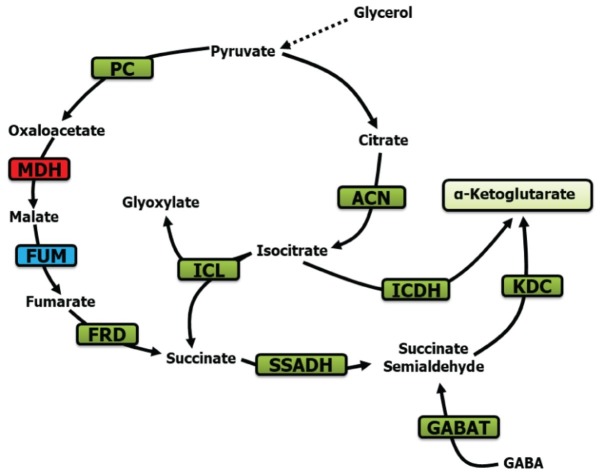
Metabolic scheme contributing to KG production. PC, pyruvate carboxylase; MDH, malate dehydrogenase; ACN, aconitase; FUM, fumarase; FRD, fumarase reductase; SSADH, succinate semialdehyde dehydrogenase; ICDH, isocitarate dehydrogenase; ICL, isocitrate lyase; KDC, ketoglutarate dehydrogenase; and GABAT, GABA aminotransaminase (green, increase; blue, no significant change; red, decrease).

In conclusion, the evidence presented in this report reveals a remarkable metabolic adaptation evoked by Mn in order to transform glycerol into KG. Succinate, SSA, fumarate, and GABA are the main participants driving this biochemical phenomenon with the assistance of the enzymes ICL, FRD, SSADH, GABAT, and KDC (Figure 8). To our knowledge, this seminal observation on a metabolic network dedicated to the production of KG *via* SSA further reveals the metabolic ingenuity of microbial systems. Although the exact role of Mn and the accompanying metabolites in this metabolic reconfiguration has to await further elucidation, it is quite possible that these modulators either individually or in concert guide the metabolism of glycerol to a pathway leading to enhanced KG production, an observation that may have important implications for the biofuel industry.

## Data Availability

All datasets generated for this study are included in the manuscript.

## Author Contributions

AA contributed to the overall experimentation and writing of the manuscript. SCT and FL contributed to overall experimentation. ST designed and performed the RT-qPCR analysis. VA contributed to project conceptualization, project management, training, and writing of the manuscript.

### Conflict of Interest Statement

The authors declare that the research was conducted in the absence of any commercial or financial relationships that could be construed as a potential conflict of interest.
